# Primary gastric adenosquamous carcinoma in a Caucasian woman: a case report

**DOI:** 10.1186/1752-1947-4-351

**Published:** 2010-10-29

**Authors:** Gil R Faria, Catarina Eloy, John R Preto, Eduardo L Costa, Teresa Almeida, José Barbosa, Maria Emília Paiva, Joaquim Sousa-Rodrigues, Amadeu Pimenta

**Affiliations:** 1 General Surgery Department, Hospital de São João/Faculty of Medicine at University of Porto, Alameda Prof. Hernâni Monteiro - Porto 4200-319, Portugal; 2 Pathology Department, Hospital de São João/Faculty of Medicine at University of Porto, Alameda Prof. Hernâni Monteiro - Porto 4200-319, Portugal

## Abstract

**Introduction:**

Most gastric tumors are adenocarcinomas. Primary gastric adenosquamous carcinoma is a rare malignancy, mostly associated with Asian populations. It constitutes less than one percent of all gastric carcinomas and its clinical presentation is the same as adenocarcinoma. It occurs more frequently in the proximal stomach, usually presents with muscular layer invasion and tends to be found in advanced stages at diagnosis, with a worse prognosis than adenocarcinoma.

**Case presentation:**

We report the case of an 84-year-old Caucasian woman with an adenosquamous carcinoma extending to her serosa with lymphatic and venous invasion (T3N1M1). Nodal and hepatic metastasis presented with both cellular types, with dominance of the squamous component.

**Conclusions:**

Adenosquamous gastric cancer is a rare diagnosis in western populations. We present the case of a woman with a very aggressive adenosquamous carcinoma with a preponderance of squamous cell component in the metastasis. Several origins have been proposed for this kind of carcinoma; either evolution from adenocarcinoma de-differentiation or stem cell origin might be possible. The hypothesis that a particular histological type of gastric cancer may arise from stem cells might be a field of research in oncological disease of the stomach.

## Introduction

Primary gastric adenosquamous carcinoma is a rare malignant neoplasia [1-3]. Most cases reported in the literature refer to Asian people [4-6]. It amounts to less than one percent of all gastric carcinomas and its clinical and endoscopic findings are similar to the intestinal-type adenocarcinoma. Its male-to-female ratio is 4:1 and it peaks in the sixth decade of life, occurring, on average, earlier than sporadic adenocarcinoma [1,7].

Adenosquamous carcinoma is a mixed neoplasia (gland-like and squamous). Intestinal-type adenocarcinomas may present with variable areas of squamous differentiation. For a diagnosis, it has been established that the squamous component should be present in more than 25 percent of the tumoral sample [8].

It appears more frequently in the esophagogastric junction but, due to its proximity with esophageal mucosa, a collision tumor (squamous cell esophageal carcinoma with gastric adenocarcinoma) cannot be ruled out and the diagnosis of adenosquamous carcinoma should not be made. Adenosquamous carcinomas usually invade deep into the muscular layer, present with venous and lymphatic invasion and tend to be diagnosed later, in more advanced stages. Its biological behaviour is usually determined by the adenocarcinoma component [1,6-10].

Primary adenosquamous gastric carcinoma is extremely rare in female Caucasian patients, with no cases reported in the international literature.

## Case presentation

We report the case of an 84-year-old Caucasian woman with a previous history of arterial hypertension controlled with an angiotensin converting enzyme (ACE) inhibitor. She had no other known personal or family history. She was referred to our hospital after consulting another hospital for epigastric abdominal pain. An upper gastrointestinal (GI) endoscopy had revealed an extensive ulcer of the gastric antrum with signs of recent bleeding and the biopsy was not representative of the lesion, so the result was negative for malignant cells.

Upon arrival at our hospital, she was pale and underweight. She presented with black stools and early satiety. A hard, well-defined abdominal mass was felt in her epigastrium. Laboratory tests revealed a hemoglobin of 7.1 g/dL, so she had a blood transfusion and was admitted to our hospital. An upper GI endoscopy with biopsy was performed, again with the same results.

**Figure 1:**
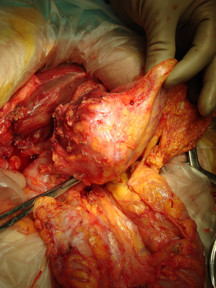
**Intra-operative - dissection from the liver surface**.

The upper gastrointestinal series was consistent with a mass in the antrum and the abdominal computed tomography (CT) scan revealed a thickening of her gastric wall and a mass without a clear interface with her liver. She underwent a sub-total distal gastrectomy with a Billroth type-2 anastomosis. Intra-operatively, she was found to have a perforated mass with extraluminal growth and adherent to the liver (Figure 1), which led to a resection of the liver parenchyma adjacent to the tumor. In the post-operative period, she developed systemic inflammatory response syndrome (SIRS) with multiple organ failure and died on the 23^rd ^post-operative day.

A macroscopic exam of the specimen revealed an ulcero-infiltrative lesion occupying most of the antrum, measuring 8 × 5 × 1.1 cm, and infiltrating her gastric wall to the serosal layer. On cutting the adherent liver fragment, a tumor was identified as a whitish nodular sub-capsular lesion of well-defined limits with a major diameter of 1.5 cm.

**Figure 2:**
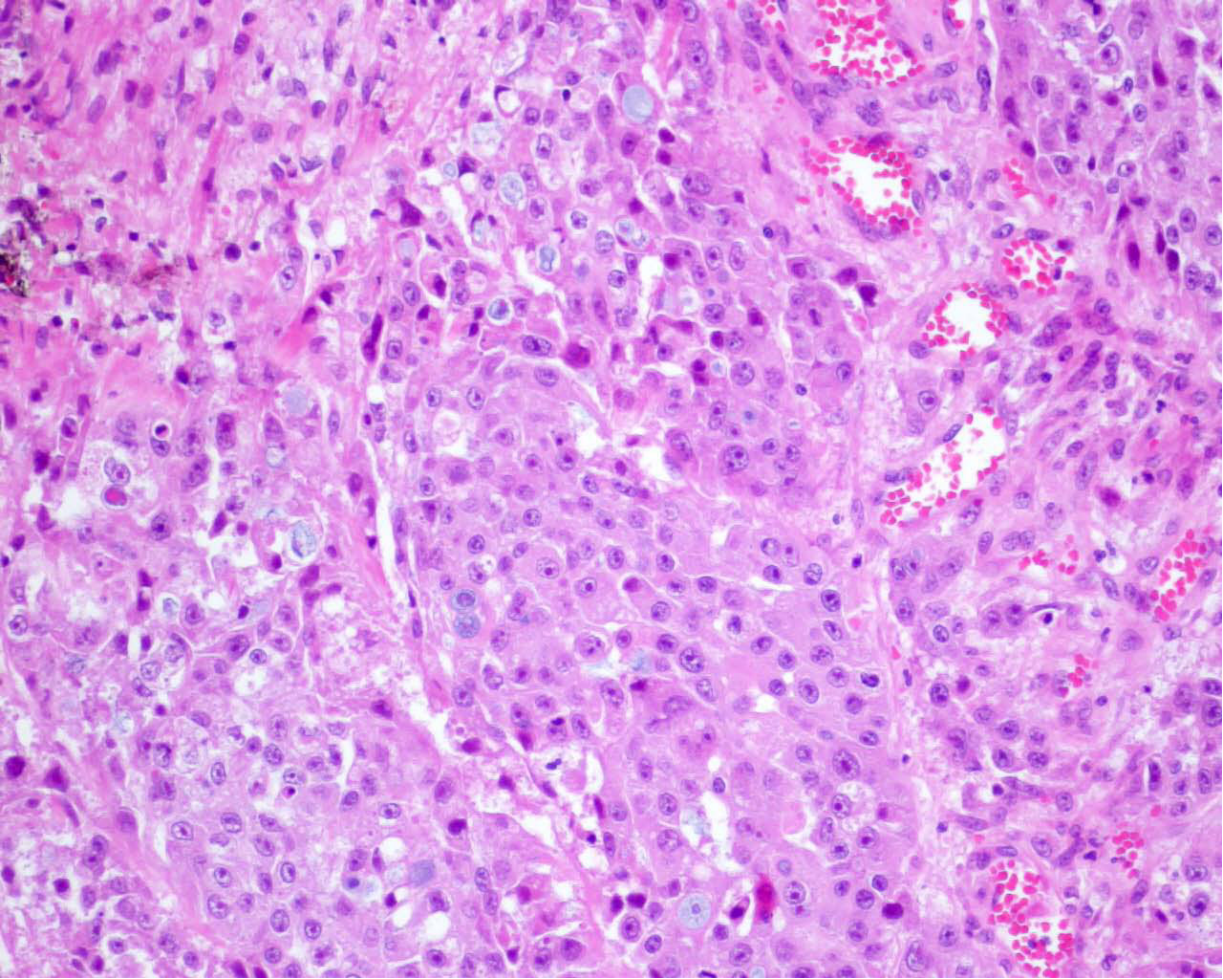
**Glandular and squamous component - adenosquamous carcinoma**.

A pathological exam revealed a poorly-differentiated malignant epithelial neoplasia of solid pattern (polygonal cells bound by Schultz bridges) and focally glandular with expansive growth (Figure 2). The lesion was perforated, with bacterial infection and significant inflammatory reaction which was responsible for the adhesion to her liver tissue. Venous and lymphatic invasion were identified, as well as peri-neural growth.

**Figure 3:**
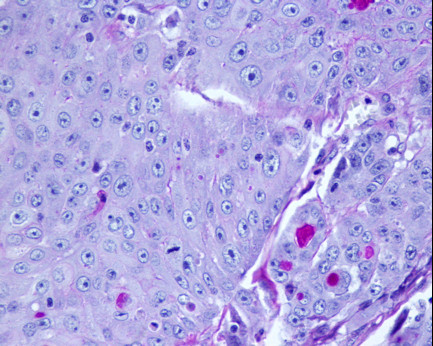
**Histology (PAS-D staining) - adenosquamous carcinoma**.

**Figure 4:**
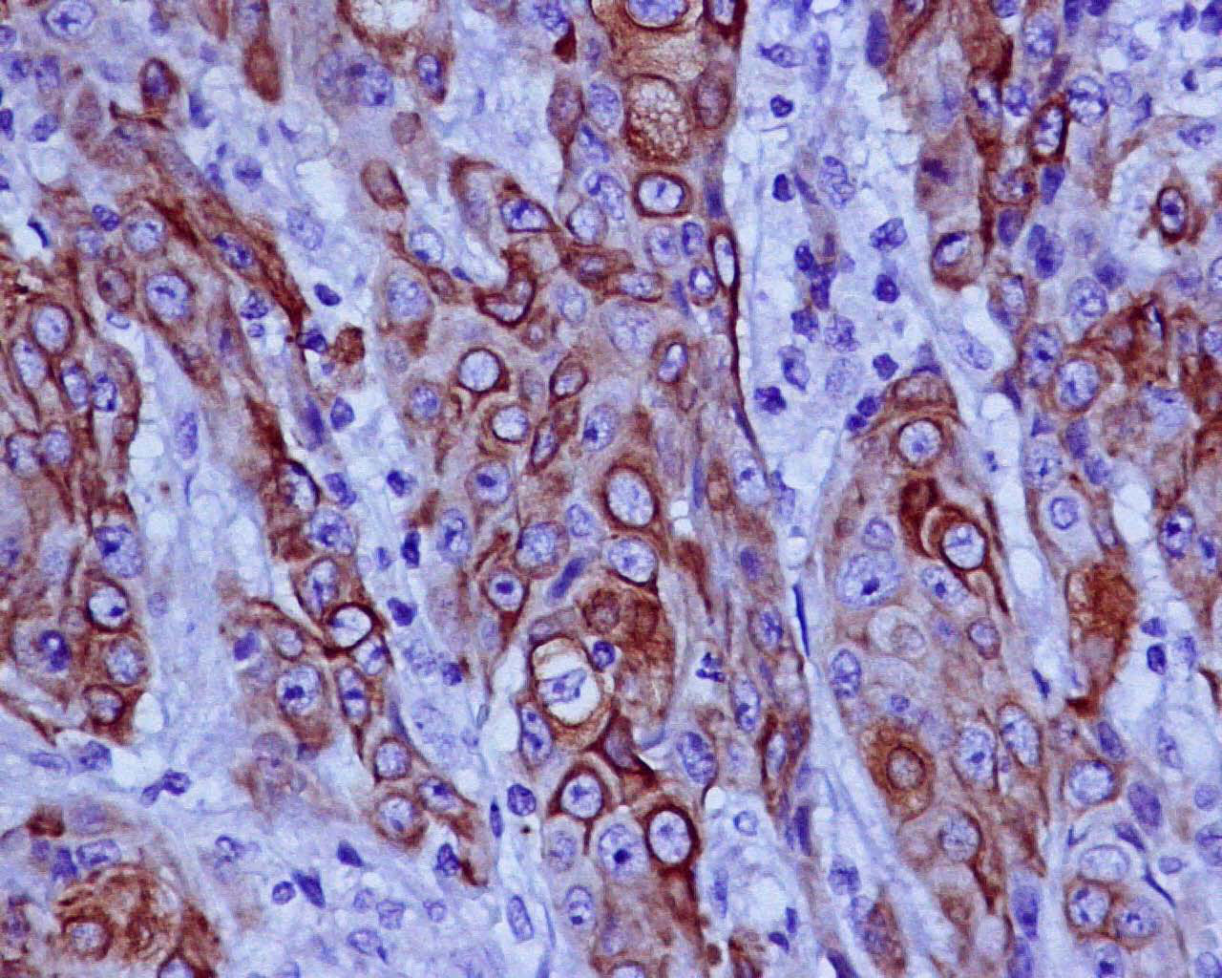
**Positive staining for squamous cell with 34βE12**.

The remaining mucosa had active atrophic chronic gastritis with intestinal metaplasia and *Helicobacter pylori *infection. No squamous metaplasia was identified. There was metastatic neoplasia in one of the eight identified lymphatic nodes, as well as in her liver fragment (without infiltration of the Gleeson capsule). A histochemical study with Periodic acid-Schiff (PAS) revealed (intracytoplasmic and extracellular) mucopolysaccharides (Figure 3). Keratinization was found in the primary tumor and on the metastasis. An immunohistochemistry panel with AE1 AE3, CAM 5.2, Chromogranin A, Sinaptophysin, HMB-45, S100 protein and c-Kit (CD117) was performed, being strong and diffusely positive for AE1 AE3 and CAM5.2 in the neoplastic cells, and negative for the remaining antibodies. A specific squamous cell staining with 34βE12 was positive (Figure 4).

**Figure 5:**
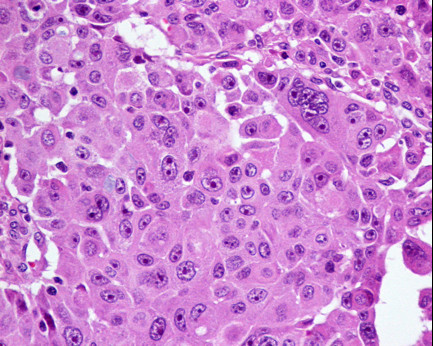
**Histology - nodal metastasis (Hematoxylin and eosin staining)**.

**Figure 6:**
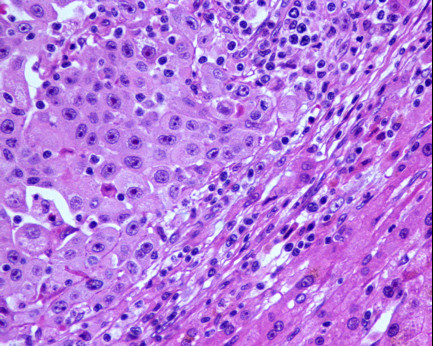
**Histology - hepatic metastasis (Hematoxylin and eosin staining)**.

This allows us to classify the tumor as a carcinoma of the gastric antrum (AE1 AE3 and CAM5.2 positivity) with a glandular component, but predominantly epidermoid (approximately 80 percent). No neuroendocrine differentiation was observed (Cromogranin A and Sinaptophysinwere negative) and gastrointestinal stromal tumor (GIST) and melanoma were excluded (c-Kit, HMB-45 and S100 protein were negative). The tumor extended to her serosa with lymphatic and venous invasion, and presented with nodal and hepatic metastasis. The nodal (Figure 5) and hepatic (Figure 6) metastasis presented both cellular lines (squamous and glandular) but the majority was occupied by the squamous component.

## Discussion

Adenosquamous carcinoma is a rare clinical entity, especially in Caucasian patients. Its occurrence is more frequent in the proximal stomach and in male patients [1,11]. For the diagnosis of a true adenosquamous carcinoma, it is necessary to confirm the presence of a mixed-pattern carcinoma (glandular and squamous component) outside the cardia, without esophageal involvement and without adenosquamous carcinoma in other organs. Besides this, it is also necessary for the squamous component to be present in over 25 percent of the tumor mass.

We report the case of an 84-year-old woman with an adenosquamous carcinoma extending to her serosa, with nodal and hepatic metastasis (T3N1M1), and also with lymphatic and venous invasion.

By reviewing her clinical records and the peri-operative findings, we can presume that her abdominal pain on presentation might be due to a perforation of the tumor next to the small curvature, contained by its close proximity with the right lobe of her liver. In spite of the negative biopsies, she was recommended for surgery due to the presence of obstructive symptoms and GI bleeding.

The tumor can be classified as T3 (according to TNM classification) as it was perforated, without direct involvement of other abdominal viscera. As it was a perforated malignant neoplasia in stage IV, a curative resection was not intended and only eight lymphatic nodes were isolated. This quantity of lymph nodes does not allow a precise staging and probably yields it under-staged. Yet, there was metastization in one of the eight nodes studied (N1). This finding is consistent with the reports in the literature; that the adenosquamous carcinoma usually behaves like an aggressive adenocarcinoma and presents with early lymphatic metastization [1,4,6,7,10].

The presence of a liver nodule not contiguous with the primary tumor was not considered as local extension of the tumor. The absence of malignant cells in the interface between her liver and her stomach, and the presence of a tumor-free Gleeson capsule, reveals the hepatic nodule to be a metastasis and not directly involved. The adherence of her liver tissue to the tumor was probably due to an inflammatory reaction after perforation. As such, the disease was considered metastized and the neoplasia classified as adenosquamous gastric carcinoma, pT3N1M1 (stage IV), which suggests a survival rate below seven percent at five years.

The prognosis for adenosquamous carcinoma is usually worse than the intestinal-type carcinoma, although its major biological determinant is the adenocarcinoma component [1,10,12]. It usually presents at a more advanced stage at diagnosis, and venous and lymphatic invasion are more often present [10,11]. The metastasis usually occurs with adenocarcinoma pattern and ultimately determines its prognosis [6,13].

Five hypotheses have been postulated for its origin: 1) metaplastic transformation of an adenocarcinoma [5,7,11,12]; 2) cancerization of metaplastic squamous cell [2,11]; 3) cancerization of ectopic squamous epithelium [14]; 4) collision of an adenocarcinoma and a squamous cell carcinoma; and 5) stem cell differentiation towards both cellular lines [1,8,12,15].

The reported findings are not in accordance with a cancerization of ectopic squamous epithelium or metaplasia, nor do they seem related to a collision carcinoma. It occurs where squamous cell metaplasia is almost non-existent (antrum), and when both cell phenotypes are present across all the tumor. There are cellular nests with both histological characteristics, and the metastization pattern is predominantly squamous.

These findings support an origin from multi-potential cells suffering double differentiation or metaplastic transformation of adenocarcinoma cells. However, there are no isolated areas of gland-like and squamous differentiation, which favours an early occurrence of both components.

The presence of nodal and hepatic metastasis as adenosquamous carcinoma with a large squamous percentage calls into question the hypothesis of the origin in a metaplastic transformation of an adenocarcinoma, as favoured by several authors [5,7,11,12]. The large squamous component in the metastasis is unusual and suggests a more aggressive behaviour of this component. This pattern suggests that the adenosquamous transformation occurred earlier in the course of the disease and did not grow over an existing adenocarcinoma.

## Conclusions

We report the case of a woman with a primary adenosquamous carcinoma of the stomach; rare in Caucasians, in women and in the gastric antrum, and unique in its metastization pattern, which was predominantly squamous. The prognosis of these tumors could not be confirmed, as she died in the post-operative period due to a septic complication.

The hypothesis that a particular histological type of gastric cancer might arise from stem cells could become a possible field of research in the oncological disease of the stomach.

## Abbreviations

ACE: Angiotensin converting enzyme; CT: Computed tomography; GI: Gastrointestinal; GIST: Gastrointestinal stromal tumor; (PAS): Periodic-Acid Schiff; SIRS: Systemic inflammatory response syndrome.

## Competing interests

The authors declare that they have no competing interests.

## Authors' contributions

GF, CE and JP wrote the article. GF, EC and JB performed the surgery. GF, JP, TA and EC were the responsible clinicians. CE and MP made the histological diagnosis. MP, JR and AP supervised the paper. All authors read and approved the final manuscript.

## Consent

Written informed consent was obtained from the patient's next-of-kin for publication of this case report and any accompanying images. A copy of the written consent is available for review by the Editor-in-Chief of this journal.
